# A Novel Antimicrobial Peptide Spampcin_56–86_ from *Scylla paramamosain* Exerting Rapid Bactericidal and Anti-Biofilm Activity In Vitro and Anti-Infection In Vivo

**DOI:** 10.3390/ijms232113316

**Published:** 2022-11-01

**Authors:** Manyu Jiang, Roushi Chen, Jingrong Zhang, Fangyi Chen, Ke-Jian Wang

**Affiliations:** 1 State Key Laboratory of Marine Environmental Science, College of Ocean & Earth Sciences, Xiamen University, Xiamen 361102, China; 2 State-Province Joint Engineering Laboratory of Marine Bioproducts and Technology, College of Ocean & Earth Sciences, Xiamen University, Xiamen 361102, China; 3 Fujian Innovation Research Institute for Marine Biological Antimicrobial Peptide Industrial Technology, College of Ocean & Earth Sciences, Xiamen University, Xiamen 361102, China

**Keywords:** mud crab *Scylla paramamosain*, antimicrobial peptide, Sapmpcin, anti-biofilm, anti-infection in vivo

## Abstract

The abuse of antibiotics leads to the increase of bacterial resistance, which seriously threatens human health. Therefore, there is an urgent need to find effective alternatives to antibiotics, and antimicrobial peptides (AMPs) are the most promising antibacterial agents and have received extensive attention. In this study, a novel potential AMP was identified from the marine invertebrate *Scylla paramamosain* and named Spampcin. After bioinformatics analysis and AMP database prediction, four truncated peptides (Spa31, Spa22, Spa20 and Spa14) derived from Spampcin were screened, all of which showed potent antimicrobial activity with different antibacterial spectrum. Among them, Spampcin_56–86_ (Spa31 for short) exhibited strong bactericidal activity against a variety of clinical pathogens and could rapidly kill the tested bacteria within minutes. Further analysis of the antibacterial mechanism revealed that Spa31 disrupted the integrity of the bacterial membrane (as confirmed by scanning electron microscopy observation, NPN, and PI staining assays), leading to bacterial rupture, leakage of cellular contents (such as elevated extracellular ATP), increased ROS production, and ultimately cell death. Furthermore, Spa31 was found to interact with LPS and effectively inhibit bacterial biofilms. The antibacterial activity of Spa31 had good thermal stability, certain ion tolerance, and no obvious cytotoxicity. It is worth noting that Spa31 could significantly improve the survival rate of zebrafish *Danio rerio* infected with *Pseudomonas aeruginosa*, indicating that Spa31 played an important role in anti-infection in vivo. This study will enrich the database of marine animal AMPs and provide theoretical reference and scientific basis for the application of marine AMPs in medical fields.

## 1. Introduction

Antimicrobial peptides (AMPs), also known as host defense peptides, are widely distributed in animals, plants, and microorganisms. It is a class of small-molecule peptides with broad antimicrobial activity and involved in physiological processes such as immune regulation, inflammation, wound repair, etc. It is a key component of host defense against bacteria, fungi, viruses, and parasites [1]. Most AMPs are cationic peptides, containing 2 to 13 positive charges, with hydrophobic residues in the majority [2]. In addition to natural sources, AMPs can also be obtained through various computational algorithm design methods and synthesis strategies. As reported, a total of 23, 253 AMPs were collected in LAMP2 database, including 7824 natural AMPs and 15, 429 synthetic AMPs [3]. Some of these small-molecule peptides are derived from the screened or optimized sequences of natural AMPs, also known as innate defense regulator peptides (IDRPs) [4], and some are a new sequence directly designed and synthesized based on physicochemical properties of amino acid (aa) sequences. For example, IDR-1018, based on Bac2A (RLARIVVIRVAR, a linear derivative of bovine cathelicidin), can effectively induce chemokine response and inhibit LPS-induced inflammatory responses, thereby killing pathogens such as *Staphylococcus aureus* and *Pseudomonas aeruginosa* [5]. AMPs derived from LL-37 or truncated peptides of natural AMPs centrocin 1 from sea urchin have shown activity against *S. aureus* and multidrug-resistant bacteria [6]. Sphistin (38 amino acids from the N-terminus of histone H2A of mud crab *Scylla paramamosain*) and its truncated peptide Sph_12–38_ showed potent antibacterial activity against *P. aeruginosa* [7].

No matter whether they are natural or synthetic AMPs, much research has shown that in addition to antibacterial functions, AMPs have antiviral, anticancer, anti-parasite, and immunomodulatory functions. For example, PR-39, a small cationic AMP rich in proline and arginine with 39 aa in length, 11 positive charges, and 20% hydrophobicity, which was first discovered in porcine intestinal cell lysates, plays an important role in innate and adaptive immunity and has a variety of functions (antibacterial, immune regulation, tissue repair, anti-apoptosis, and pro-angiogenesis, etc.) [8]. Compared with single-target antibiotics, AMPs have the advantages of diverse mechanisms of action, rapid pathogen killing, good biocompatibility, and difficulty in developing drug resistance etc., making them the most promising antibacterial agent to replace antibiotics, and have good application prospects in agriculture, aquatic products, food, and medicine in the future [9].

In recent years, in order to solve the problem of frequent bacterial diseases in aquaculture animals, the extensive use or abuse of antibiotics has led to the continuous acquisition of new genetic traits or resistance genes by bacteria, thus allowing bacteria to survive and proliferate in stressful environments. The increase of bacterial resistance results in problems, such as antibiotic residues in aquatic products and antibiotic pollution in aquaculture environment, which seriously threatens the development of aquaculture and human health [10]. Therefore, it is an urgent need to find effective alternatives to antibiotics. Marine animals live in complex aquatic environments rich in microorganisms, eutrophication, and pollutants, especially invertebrates, relying solely on innate immunity to maintain health and survival. AMPs are important immune effectors that help the body defend against the invading pathogens. With the deepening of research, it has been reported that the AMPs produced in invertebrates have the characteristics of diversity (such as crustacean crustins, anti-lipopolysaccharide factors, penaeidins, etc.) and specificity (many novel AMPs have been identified) compared with vertebrates, which may be their unique strategy for adapting to a threatened living environment. Therefore, marine animals have become an important resource for the development of new antibacterial and antifungal agents in the future. However, the identification of marine animal AMPs is still insufficient, and deserves more attention and investigation.

In this study, based on transcriptome data previously constructed in our laboratory [11], we obtained a novel antimicrobial peptide, named Spampcin, from the marine mud crab *Scylla paramamosain*. Its full-length cDNA was obtained by RACE PCR and its expression profile in tissues of adult crabs and immune response patterns in the hepatopancreas of crabs after bacterial stimulation were analyzed. Furthermore, based on the physicochemical properties of AMPs and AMP database prediction, four truncated peptides derived from Spampcin were designed and chemically synthesized, and their antibacterial and antifungal activities were determined. Spa31 was selected to further explore its antibacterial mechanism in vitro and reveal its anti-infection effect in vivo using a zebrafish bacterial infection model.

## 2. Results

### 2.1. Sequence Analysis and Expression Profiles of Spampcin Gene

The full-length cDNA sequence of Spampcin is 1040 bp with an open reading frame (ORF) of 462 bp (primers used for cDNA cloning were listed in Table S1), encoding 153 amino acids, without a signal peptide (GenBank accession NO. MW388709) (Figure S1A). The mature peptide of Spampcin has a molecular weight of 16.1 kDa and an estimated isoelectric point (pI) of 9.44, which showed high possibility to be classified as an AMP (Table 1).

Quantitative real-time PCR (qPCR) results showed that the Spampcin gene was widely distributed in different tissues of female adult crabs, with relatively high expression in the ovaries (Figure S1B) (primers used for qPCR were listed in Table S1). In male adult crabs, the Spampcin gene was predominantly expressed in the gills (Figure S1C). We further investigated the expression profiles of the Spampcin gene in the hepatopancreas of male adult crabs following a bacterial challenge (Vibrio alginolyticus or *S. aureus*). The results showed that the Spampcin gene expression was significantly induced at 3 h after V. alginolyticus infection. In response to an *S. aureus* challenge, the Spampcin gene was downregulated at 3 h and upregulated at 24 h (Figure S1D,E).

### 2.2. Truncated Peptides Derived from Spampcin Show Potent and Broad-Spectrum Antimicrobial Activity

**Figure 1:**
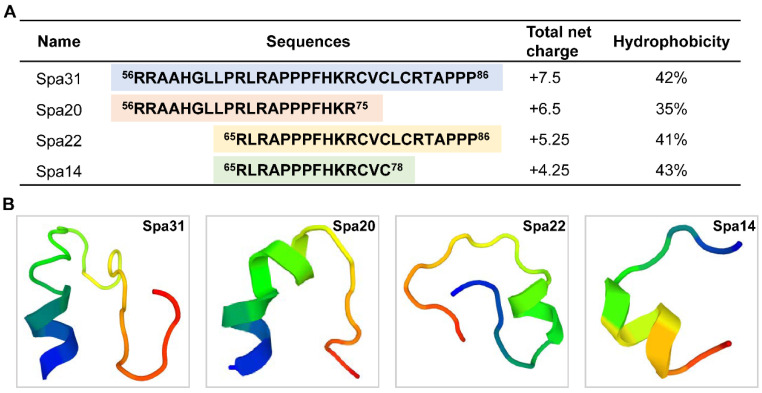
Basic information of the four synthetic peptides. (**A**) Peptide sequences, total net charge and hydrophobicity of Spa31, Spa20, Spa22, and Spa14. (**B**) The predicted 3D structures of Spa31, Spa20, Spa22, and Spa14 using the PepFold tool.

Based on bioinformatics analysis, physicochemical properties, and AMP database predictions, a truncated peptide containing 31 amino acids derived from the mature peptide of Spampcin (a region from 56th to 86th) was found to have a high AMP potential (Table 1), named Spampcin_56–86_ (Spa31 for short). The net charge and hydrophobicity of Spa31 is +7.5 and 42% (Figure 1A). Spa31 was then chemically synthesized and subjected to antimicrobial activity evaluation. It was found that Spa31 exhibited potent antibacterial and antifungal activity against Gram-negative bacteria (such as *P. aeruginosa*, Escherichia coli, Acinetobacter baumannii), Gram-positive bacteria (such as *S. aureus*, Listeria monocytogenes, Enterococcus faecalis) and fungi (such as Fusarium graminearum, Fusarium solani) with MIC values ranging from 1.5–3 μM and MBC or MFC values lower than 6 μM (Table 2).

Considering subsequent applications, as shortening the length of synthetic peptides can effectively reduce costs, we sought to investigate the AMP potential of smaller peptides derived from Spa31. Therefore, three truncated peptides were further designed and synthesized, named Spampcin_56–75_, Spampcin_65–86_, Spampcin_65–78_ (Spa20, Spa22, and Spa14 for short, respectively) according to the location and number of amino acids contained. Their AMP prediction results were shown in Table 1 and the specific sequences, locations, and physicochemical parameters (net charge and hydrophobicity) were summarized in Figure 1A. The results of antimicrobial assay showed that Spa20 and Spa22 also had broad-spectrum antibacterial activities (Table 2). Among the three peptides (Spa20, Spa22, and Spa14), Sap22 had the most potent activity against Gram-positive and Gram-negative bacteria with MBC values ranging from 3–12 μM, while the activity of Spa14 was weakest, and only showed a bit activity against L. monocytogenes and A. baumannii (Table 2). All three of the peptides showed similar antifungal activity against F. graminearum (MFC value of 12–24 μM) (Table 2). In particular, Spa22 exhibited the strongest fungicidal activity against Crytococcus neoformans (MFC value of 3–6 μM) (Table 2). The predicted 3D structures of the four peptides were shown in Figure 1B, and all of them contained mainly helical and irregularly coiled structures.

**Figure 2:**
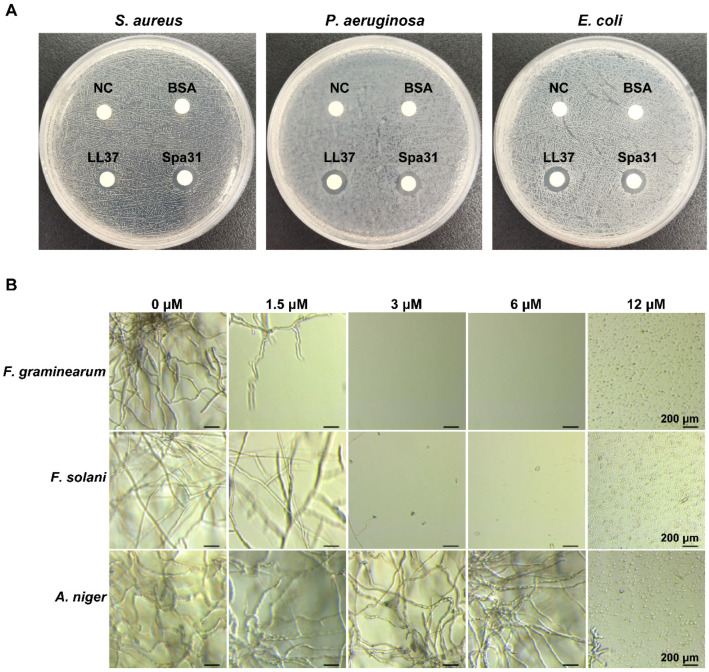
Inhibitory effect of Spa31 treatment on bacterial and fungal spore germination. (**A**) Zone of inhibition against bacteria (including *S. aureus*, *P. aeruginosa*, and *E. coli*) after treatment with 20 μL of Spa31, LL-37, or BSA at a concentration of 1 μg/μL. NC represented the negative control group treated with Milli-Q water. (**B**) Effects of Spa31 on spore germination of *F. graminearum*, *F. solani*, and *Aspergillus niger* at different concentrations (1.5, 3, 6, and 12 μM).

Among the four peptides (Sap31, Spa20, Spa22, and Spa14), Spa31 exhibited the most potent antibacterial activity against a variety of clinical pathogens, therefore, it was selected for further investigation. The zone of inhibition assay confirmed that Spa31 had significant antibacterial activity against *S. aureus*, *P. aeruginosa*, and *E. coli* (Figure 2A). The diameter of the blank antimicrobial susceptibility disc is 6.19 mm. The inhibition zone diameters of Spa31 against *S. aureus*, *P. aeruginosa*, and *E. coli* were 10.51 ± 0.07 mm, 10.06 ± 0.06 mm, and 10.57 ± 0.03 mm, respectively, corresponding to 9.50 ± 0.12 mm, 9.87 ± 0.07 mm, and 10.27 ± 0.03 mm for LL-37. In addition, it was observed that 3 μM of Spa31 could effectively inhibit fungal spore germination of F. graminearum and F. solani (Figure 2B).

### 2.3. Effective Bactericidal Activity of Spa31 against S. aureus and P. aeruginosa

**Figure 3:**
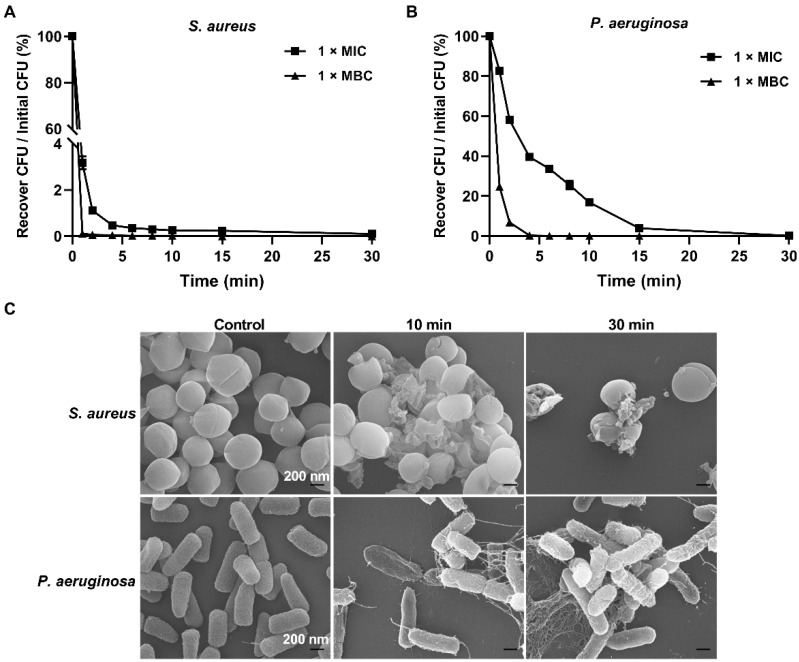
Bactericidal kinetics and SEM observations of Spa31-treated *S. aureus* and *P. aeruginosa*. Time-killing curves of *S. aureus* (**A**) and *P. aeruginosa* (**B**) treated with 1 × MIC and 1 × MBC concentrations of Spa31. (**C**) The effect of Spa31 on *S. aureus* and *P. aeruginosa* observed by SEM at 10 min and 30 min.

The bactericidal efficiency of Spa31 was evaluated using a time-killing kinetic assay. The results showed that when incubated with *S. aureus* and *P. aeruginosa*, 6 μM Spa31 rapidly killed more than 99.9% of *S. aureus* within 2 min and eliminated 99.9% of *P. aeruginosa* within 6 min (Figure 3A,B).

SEM observations revealed that after Spa31 treatment, *S. aureus* exhibited cell rupture, disruption of membrane integrity, and even leakage of cellular contents (Figure 3C); the surface of *P. aeruginosa* was obviously rough, filamentous substances appeared, and finally the cell contents flowed out (Figure 3C).

**Figure 4:**
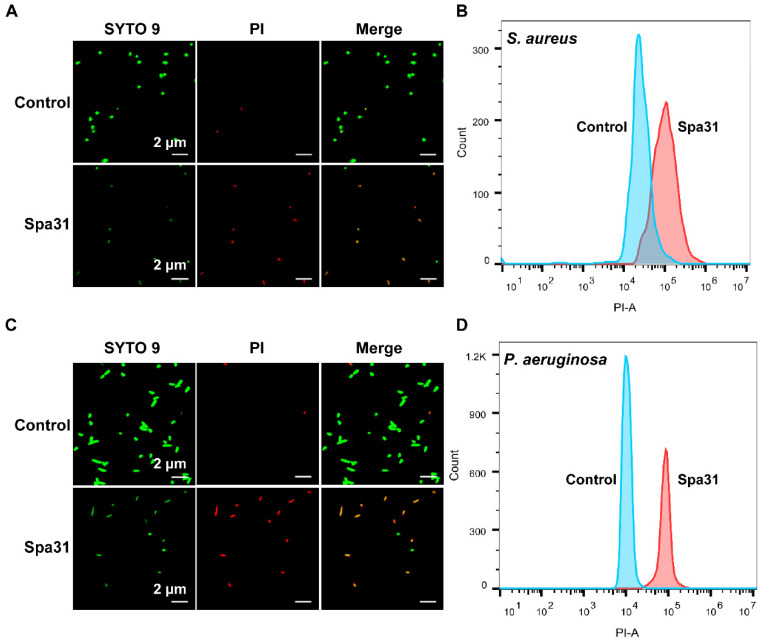
The permeability of Spa31 on the bacterial inner membrane of *S. aureus* and *P. aeruginosa*. The fluorescence signals of SYTO 9 (green) and PI (red) in *S. aureus* (**A**) and *P. aeruginosa* (**C**) observed by confocal laser scanning microscope. The fluorescence intensities of PI in *S. aureus* (**B**) and *P. aeruginosa* (**D**) detected by flow cytometry.

SYTO 9 and PI staining analysis further confirmed the disruption of bacterial membrane integrity by Spa31. As shown in Figure 4A,C, the fluorescence signal of PI was obviously enhanced after Sap31 treatment compared with the control group, and together with the evidence provided by flow cytometry (Figure 4B,D) indicated that Spa31 significantly increased the bacterial inner membrane permeability of *S. aureus* and *P. aeruginosa*.

### 2.4. Antibacterial Mechanism of Spa31 against P. aeruginosa

**Figure 5:**
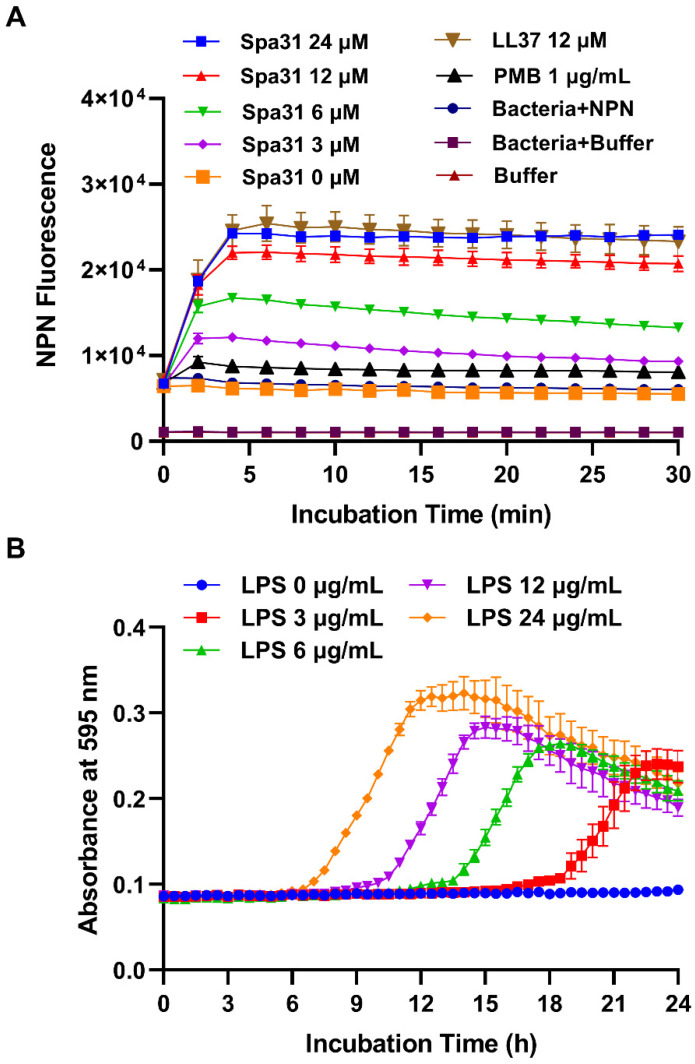
The outer membrane permeability of Spa31 against *P. aeruginosa* and the interaction between Spa31 and *P. aeruginosa*-derived LPS. (**A**) NPN fluorescence detection after treatment with different concentrations of Spa31. LL-37 and PMB treatment groups were used as controls. (**B**) Effect of addition of *P. aeruginosa*-derived LPS on the antibacterial activity of Spa31 against *P. aeruginosa*.

To further investigate the antibacterial mechanism of Spa31 against *P. aeruginosa*, NPN staining assay was conducted to reveal the effect of Spa31 on the outer membrane of *P. aeruginosa*. As shown in Figure 5A, as the concentration of Spa31 treatment increased, the NPN fluorescence intensity was obviously enhanced. LL-37 treatment (12 μM) also had similar results to the 24 μM Spa31 treatment group, while the antibiotic PMB treatment showed low fluorescence intensity (Figure 5A). The results indicated that Spa31 could cause disruption of the outer membrane of *P. aeruginosa* in a dose-dependent manner.

Whether Spa31 interacts with bacterial surface components such as LPS prior to outer membrane penetration deserves exploration. Without the addition of LPS, Spa31 at a concentration of 1 × MIC completely inhibited the growth of *P. aeruginosa* within 24 h (Figure 5B). However, when 3 μg/mL *P. aeruginosa*-derived LPS was added to the medium, *P. aeruginosa* began to grow exponentially at approximately 21 h of incubation (Figure 5B). As the increase of the concentration of LPS (6, 12, 24 μg/mL), the exponential growth time decreased in a dose-dependent manner (Figure 5B).

**Figure 6:**
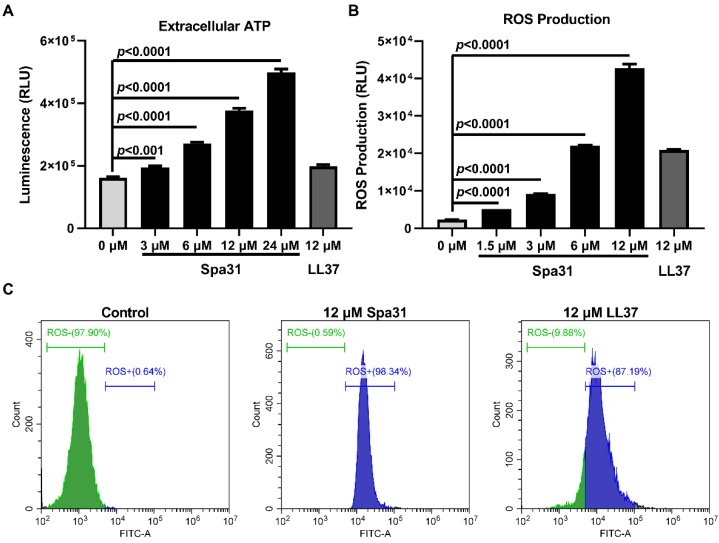
Effects of Spa31 on ATP release and ROS production in *P. aeruginosa*. (**A**) Detection of extracellular ATP in *P. aeruginosa* after Spa31 treatment. (**B**) The fluorescence intensities of DCFH-DA in *P. aeruginosa* after Spa31 treatment was analyzed by microplate reader (**B**) and flow cytometry (**C**). LL-37 treatment group was used as a control.

The results have demonstrated that Spa31 disrupted the inner and outer membranes of *P. aeruginosa*, and we also found that Spa31 treatment resulted in the release of ATP (Figure 6A) and the production of ROS (Figure 6B) in a dose-dependent manner. Flow cytometry analysis showed that a 12 μM Spa31 treatment caused 98.34% ROS production, compared with 0.64% and 87.19% in the control and 12 μM LL-37-treated groups, respectively (Figure 6C).

### 2.5. Anti-Biofilm Activity of Spa31 against P. aeruginosa

**Figure 7:**
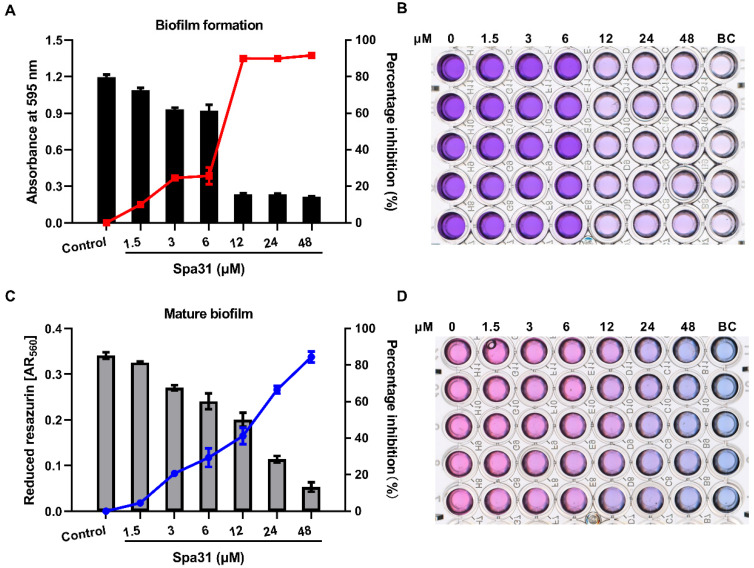
Anti-biofilm activity of Spa31 against *P. aeruginosa*. The inhibitory effect of Spa31 on biofilm formation (**A**,**B**) or mature biofilm (**C**,**D**) of *P. aeruginosa*.

Biofilm formation is a strategy that is well developed for *P. aeruginosa* to adapt to the stresses of environment and antibiotics, which are more difficult to eliminate. Since Spa31 exerted rapid and efficient bactericidal activity against planktonic *P. aeruginosa*, the effect of Spa31 on *P. aeruginosa* biofilm formation or mature biofilm was also evaluated. Spa31, at a concentration of 1.5 μM, significantly inhibited biofilm formation by approximately 10% (*p* < 0.001) (Figure 7A,B). Especially, when the concentration of Spa31 reached 12 μM, the biofilm mass reduced by 90% (*p* < 0.0001) (Figure 7A,B). Furthermore, Sap31 could eradicate mature biofilms of *P. aeruginosa* in a dose-dependent manner, with an eradication rate of 84.4% at 48 μM (*p* < 0.0001) (Figure 7C,D).

### 2.6. In Vitro Determination of Spa31 Properties and In Vivo Anti-P. aeruginosa Infection Analysis

**Figure 8:**
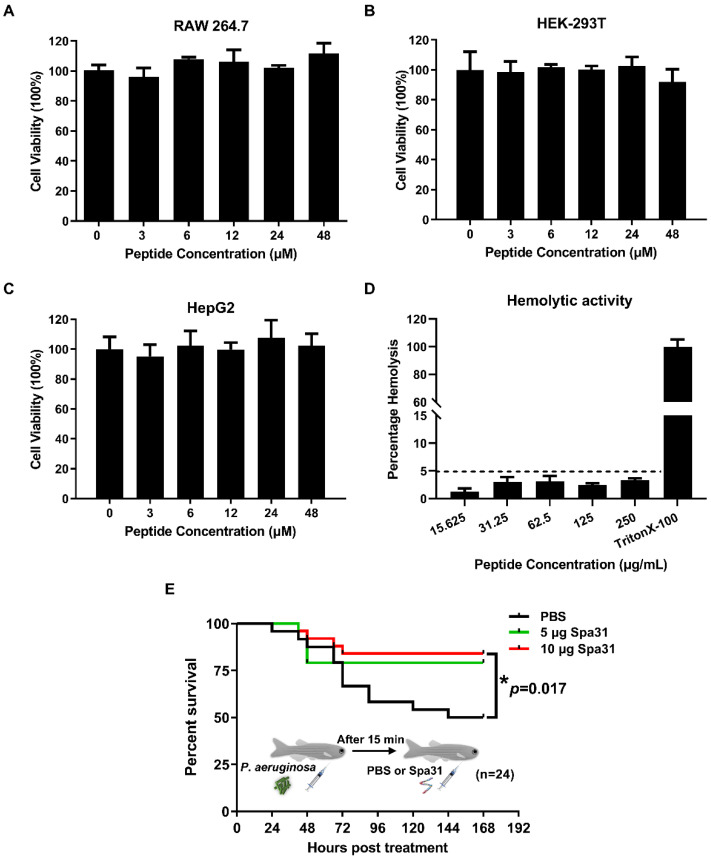
In vitro cytotoxicity and hemolytic activity and in vivo anti-*P. aeruginosa* infective effect of Spa31. (**A**–**C**) The effect of Spa31 on cell viability of RAW264.7, HEK-293T, and HepG2 cell lines. (**D**) The hemolytic activity of Spa31 on mouse erythrocytes. TritonX-100 treatment was used as the control. (**E**) The survival curve of *P. aeruginosa*-infected zebrafish treated with 5 μg and 10 μg Spa31.

In vitro determination of various properties of AMPs, including cytotoxicity, hemolytic activity, thermostability, and ionic tolerance, will provide a scientific basis for their future applications. In this study, we found that Spa31 was not toxic towards several cell lines (including RAW264.7, HEK-293T, and HepG2 cells), even at a concentration of 48 μM (Figure 8A–C). Furthermore, Spa31 also exhibited no hemolytic activity on mouse erythrocytes, even at a high concentration of 250 μg/mL with the hemolysis rates lower than 5% (Figure 8D). In addition, it had good thermal stability, and its antibacterial activity against *S. aureus*, *P. aeruginosa*, and *E. coli* was still maintained after a 30 min treatment in a 100 °C water bath (Figure S2A–C). It exhibited a certain ionic tolerance, and the addition of 10 mM to 80 mM NaCl did not affect its antibacterial activity (Figure S2D–F).

To investigate whether Spa31 has an anti-infective effect in vivo, the zebrafish *Danio rerio* is commonly used as an animal model. It was found that 10 μg Spa31 treatment significantly improved the survival of zebrafish infected with *P. aeruginosa* (*p* = 0.017) (Figure 8E). After 168 h of treatment, the PBS group had a survival rate of 50%, compared with 79% in 5 μg Spa31 treatment group or 85% in 10 μg Spa31 treatment group (Figure 7E).

## 3. Discussion

In studying the marine invertebrate *S. paramamosain*, many efforts and exciting progress have been made in discovering new AMPs. For example, the scygonadin gene and its homolog SCY2-5, which were all predominantly expressed in the ejaculatory ducts of adult male crabs, have been shown to participate in fertilization and reproduction immunity of mud crabs [12,13,14]. Interestingly, a further in-depth study found that the SCY2-interacting protein Scyreprocin, screened using yeast two-hybrid technology, has also been proven to be an AMP with broad-spectrum antibacterial, antifungal, and anti-biofilm activities [15]. Recently, the newly identified AMP Sparanegtin, which was highly expressed in the testis, was responsive to LPS and *V. alginolyticus* stimulation, and exerted immunomodulatory effects to improve the survival of *V. alginolyticus*-infected crabs [16]. Similarly, in the study, Spampcin was also a novel functional gene, which was widely distributed in the tissues of adult crabs and was significantly regulated by *V. alginolyticus* and *S. aureus* challenge, indicating that it also played a role in the immune response of *S. paramamosin*. Further studies are needed to obtain its recombinant protein and confirm its immune-related functions in vitro and in vivo. Other novel AMPs, such as Sparamosin_26–54_ [17], Sphistin [7,18], SpHyastatin [19], Sp-NPFin [20], etc., including the above AMPs as mentioned, all play an important role in the innate immunity of *S. paramamosin* and have good prospects for future applications in aquaculture and medicine. Here, in this study, another novel functional gene with high AMP potential, named Spampcin, was characterized from *S. paramamosain* and its derived truncated peptide Spa31 had been confirmed to exhibit broad-spectrum and potent antibacterial and antifungal activity, and several shortened Spa31-derived peptides (Spa20, Spa22, and Spa14) also had certain antimicrobial activity. Further analysis of the in vitro properties, antibacterial mechanism, and in vivo anti-infection effect of Spa31 will lay the foundation for the application of marine animal-derived AMPs in the future.

At present, there are various databases that provide computational tools for the prediction of AMPs, such as CAMP_R3_, AntiBP2 [21], ClassAMP [22], APD3 [23], AMPer [24], etc., and their predictions are based on different algorithms. For example, in the CAMP_R3_ database, according to the conserved sequence signatures of AMPs (captured as patterns and Hidden Markov Models), 4 different prediction methods are involved, including support vector machine (SVM), random forest (RF), artificial neural network (ANN), and discriminant analysis (DA), which have become useful tools to discover and design novel AMP candidates [25]. In the study, we designed and synthesized four truncated peptides with high AMP potential derived from Spampcin (Spa31, Spa20, Spa22, and Spa14) through CAMP_R3_ database prediction, and compared and analyzed their antibacterial activities.

Previous studies have demonstrated that peptide hydrophobicity, amphipathicity, helicity, and net charge number are important factors affecting the biological activities (such as antimicrobial activity, hemolytic activity, etc.) of AMPs [26,27,28,29]. Within a certain range, the higher the positive charge and hydrophobicity, the stronger the antibacterial activity of α-helical AMPs [26,27]. However, beyond the optimum window, a decrease in antimicrobial activity and an increase in hemolytic activity were observed [26]. In particular, the net charge and hydrophobicity are inseparable from the secondary structures of AMPs (such as α-helical, β-sheet, linear/cyclic, irregularly coiled or mixed extended structures) [30]. Many efforts have been made to improve the antibacterial activity, enhance the stability, and reduce the hemolytic activity of AMPs, such as increasing the number of positively charged residues on the polar face [27], replacing L-type amino acids with D-enantiomers [29], incorporating unnatural amino acids (such as L-Dab, L-Dap) [31] or “specificity determinants” (such as Lys residues) [28], and multimerization of AMP [32], etc. [30]. In the study, it was found that the three peptides (Spa31, Spa20, and Spa22) all had good antibacterial activities, among which Spa31 had relatively stronger antibacterial and antifungal activity with the MBC range of 3–6 μM or 6–12 μM, which had the greatest hydrophobicity (42%) and the highest positive net charge (+7.5). We speculated that their net charge and hydrophobicity might fall within the optimal range as mentioned above. In addition, the 3D structure predictions indicated that all four synthetic peptides had a mixed extended structure, including α-helix, which needs to be confirmed by further evidence (e.g., circular dichroism analysis). The differences in the second structure might be attributed to their different antibacterial activity spectrum, and the underlying mechanism deserves in-depth investigation. Among them, Spa14 only showed antibacterial activity against two bacteria, with the MBC value of 12–24 μM against *L. monocytogenes* and 48–96 μM against *A. baumannii*. However, Spa14 still had certain antifungal activity (such as *C. neoformans*, *A. niger*, *F. graminearum*, and *F. solani*). Compared with other synthetic peptides, Spa14 had the shortest peptide length and the least positive charge but is similar in hydrophobicity to Spa22 and Spa31. Although Spa14 has weak antibacterial activity, it may still be used as an antifungal agent targeting fungi in the future. For most bacteria tested, Spa31 is always the best choice, but for some bacteria (such as *A. baumannii*, *C. neoformans*), the shortened peptides Spa20, Spa22, or Spa14 can be used to reduce application costs. Therefore, they all have potential application prospects.

Spa31 was further selected to reveal its antibacterial mechanism. We first observed the disruptive effect of Spa31 on bacterial morphological structure by SEM and confirmed the penetration of Spa31 into the bacterial outer and inner membrane by NPN, SYTO9, and PI staining using flow cytometry and confocal microscopy. Most AMPs can directly damage bacterial cell membranes [33,34,35]. For example, AMPR-11 causes changes in cell membrane permeability and cell rupture in carbapenem-resistant *P. aeruginosa* and methicillin-resistant *S. aureus* [36]. Similarly, after Spa31 treatment, the results showed that the inner membranes of *S. aureus* and *P. aeruginosa* were damaged, and a large amount of PI dye entered the cells. Both bacteria aggregated, had rough surface, shrank, and the contents flowed out, and filamentous substances appeared on the surface of *P. aeruginosa*. The human cathelicidin LL-37 is commonly used as a positive control, which has been demonstrated to exert antibacterial activity through disrupting bacterial membranes [37]. It is well known that the outer membrane of Gram-negative bacteria is their first barrier against environmental stress and is also the target of many antimicrobial agents. LPS is an important component of the outer membrane of Gram-negative bacteria and is negatively charged. Studies have shown that cationic AMPs, such as SNAPP, Brevicidine, and Laterocidine [38,39], can bind to LPS through electrostatic interactions, which is the first critical driver of their ability to target cell membranes. When AMPs accumulate to a threshold concentration on the bacterial surface, they can cause cell membrane lysis, thereby killing bacteria [40,41]. In this study, the addition of LPS reduced the anti-*P. aeruginosa* activity of Spa31 in a concentration-dependent manner, which indirectly indicated that Spa31 (also as a cationic AMP) could interact with LPS, and eventually disrupt the bacterial outer cell membrane (enhanced NPN fluorescence intensity was observed). After the permeability of the bacterial cell membrane changes, intracellular substances are released or leaked out of the cell. ATP is an energy substance produced by bacterial intracellular metabolism, and the release or leakage of this substance will affect the life activities of bacteria. Following AS-hepc3_(48–56)_ or α-helical AMP II treatment, the content of ATP in the bacterial extracellular environment was significantly increased in a concentration-dependent manner [42,43]. Likewise, in the study, Spa31 also caused an increase in extracellular ATP levels.

In addition, it is generally believed that excess ROS (reactive oxygen species) can rapidly oxidize various biological macromolecules such as DNA, proteins, and lipids in cells, thereby causing damage to organisms [44]. Many studies have shown that AMPs can induce excessive ROS production, leading to bacterial oxidative damage. For example, Cathelicidin can significantly increase ROS levels and induce the expression of *E. coli* ArcA, OxyR, gshA, and other genes, which play key roles in resistance to ROS damage [45]. In the study, 12 μM Spa31 treatment resulted in a 98.34% increase in ROS compared to the control group (0.64%) and LL-37 treatment group (87.19%), indicating that Spa31 caused severe oxidative stress in *P. aeruginosa*. This might be another strategy for Spa31 to effectively kill bacteria, in addition to disrupting cell membranes. Notably, some proline- and arginine- rich AMPs, such as A3-APO and Arasin 1, have multiple antibacterial mechanisms, and they interact with intracellular targets. For example, they bind to the heat shock protein DnaK, which causes protein misfolding and aggregation in cells, inhibition of biosynthesis, etc., and eventually leads to bacterial death [46]. Whether Spa31 has a similar bactericidal mechanism deserves further study. Furthermore, it is worth mentioning that the rapid bactericidal process of AMPs and the mechanism of action on cell membranes make it difficult to induce bacterial resistance [43,47]. In the study, Spa31 was able to kill *S. aureus* and *P. aeruginosa* within minutes and had anti-biofilm activity against *P. aeruginosa*. As reported, the antibiofilm mechanism of AMPs varies and involves different biofilm formation processes. For example, LL-37 can inhibit the formation of *P. aeruginosa* biofilm by reducing bacterial adhesion and disrupting the quorum-sensing systems [48]. Human β-defensin 3 regulated the expression of biofilm formation-related genes, such as binding protein transport genes [49,50]. Nisin A and esculentin(1–21) have been demonstrated to eliminate mature biofilms through disrupting bacterial membranes [51,52]. Peptide G3 may be involved in the entire biofilm formation processes, such as inhibiting bacterial attachment, destroying mature biofilm structure through interacting with extracellular DNA, and dispersing it [53]. Similarly, Spa31 could inhibit both *P. aeruginosa* biofilm formation and mature biofilm, and whether it exerts its antibiofilm effect through a mechanism similar to the aforementioned AMPs needs to be confirmed by further studies. In addition, whether Spa31 and the other three peptides exhibit antibacterial activity against clinical drug-resistant bacteria and does not induce bacterial resistance requires further evidence.

Due to the potential application value of Spa31, it is necessary to reveal whether its antibacterial activity is affected by various factors (such as temperature, cation, pH, etc.). BAMP derived from *Bacillus paralicheniformis* has a wide temperature (4–125 °C) and pH (2.0–9.0) tolerance, and is not easily degraded by trypsin, pepsin, and protease K. [54]. The AMP HSPE3 retained its antibacterial activity against *Bacillus cereus* regardless of high temperature steam heating or dry heating [55]. In this study, the results showed that Spa31 maintained antibacterial activity against *S. aureus*, *P. aeruginosa*, and *E. coli* after 30 min of treatment in boiling water, indicating it had good thermal stability (Figure S2A–C). Furthermore, the electrostatic attraction between cationic AMPs and negatively charged bacterial surfaces was found to be easily shielded or disrupted in the presence of high concentrations of cations [56]. Under high salinity conditions, many AMPs have been reported to be inactivated, such as human β-defense-1 (inactivated by 125 mM of NaCl) and LL-37 (completely inactivated by 100 mM NaCl) [57,58,59]. However, some AMPs have good ionic tolerance. For example, human β-defensin 3 (hBD3) has antibacterial activity against various pathogens in the presence of 150 mM Na^+^. Among them, the C-terminal “RRKK” motif of hBD3 is thought to play a role in its cation tolerance mechanism [60]. In the study, Spa31 also showed a certain ion tolerance against *S. aureus*, *P. aeruginosa*, and *E. coli* under the condition of 80 mM Na^+^ (Figure S2D–F). We speculate that Spa31 contains a high positive charge and binding activity to LPS, which might contribute to its resistance to cations. Taken together, the novel AMP Spa31 has good in vitro thermal stability and ion tolerance, laying a foundation for future application in food preservation, antibacterial agents, or feed additives. In addition, the stability of Spa31 under other environmental conditions, such as pH, serum, and various enzymes still needs to be further clarified.

It is believed that AMPs may have strong hemolytic activity due to their high positive charge number, which limits their application in animals. It has been reported that peptides with more than 9 positive charges can cause damage to blood cells. For example, AMP MAP-36 with 14 positive charges produces hemolysis at a concentration of 4 μM, while AMP RI12 with 6 positive charges shows hemolytic activity at a concentration of 128 μM [61]. In this study, Spa31 possessed biocompatibility and did not cause hemolysis of mouse erythrocytes at concentrations up to 250 μg/mL (72 μM), whose therapeutic index would exceed 24 against *P. aeruginosa* (the MIC value was 3 μM) (calculated by the ratio of hemolytic activity and MIC as described previously [29]). Meanwhile, the cytotoxicity of Spa31 was evaluated, and it did not affect cell viability under the concentration of 48 μM, which laid a foundation for subsequent development and application. Based on this, an in vivo model of bacterial infection in zebrafish *D. rerio* was established in this study to explore whether Spa31 had an anti-infective effect in vivo. The results showed that injecting a certain amount of Spa31 (10 μg per fish) significantly improved the survival rate of *D. rerio* infected with *P. aeruginosa*, indicating that Spa31 had a significant immunoprotective effect in vivo. The doses of Spa31 used in zebrafish were selected with reference to those used in actual clinical applications [62] and in mouse models [43,63,64] (5–20 mg/kg). Other AMPs have also used zebrafish infection models to evaluate their in vivo effects, such as Scyreprocin, epinecidin-1, and fish-derived cathelicidins, with effect doses of 1–8 μg, 1–10 μg, and 10 μg per fish, respectively [15,65,66]. We believe that the 10 μg Spa31 dose determined in this study provides a convincing reference dose for future clinical applications. Taken together, in this study, the preliminary analysis of the immune effect of the Spa31 in vitro and in vivo will lay a foundation for further in-depth discussion of the molecular mechanism of this AMP and provide reference for the clinical application of this peptide in the future.

## 4. Materials and Methods

### 4.1. Microorganism Strains, Cell Lines, and Reagents

The strains were purchased from China General Microbiological Culture Collection Center (CGMCC), Beijing, China, including *S. aureus* (CGMCC 1.2465), *L. monocytogenes* (CGMCC 1.10753), *E. faecalis* (CGMCC 1.2135), *Enterococcus faecium* (CGMCC 1.1310), *Staphylococcus epidermidis* (CGMCC 1.4260), *P. aeruginosa* (CGMCC 1.2421), *E. coli* (CGMCC 1.2389), *A. baumannii* (CGMCC 1.6769), *C. neoformans* (CGMCC 2.1563), *F. graminearum* (CGMCC 3.4521), *F. solani* (CGMCC 3.5840), and *A. niger* (CGMCC 3.3160). The bacterial and fungal strains were cultured in nutrient broth (OXOID, UK) agar at 37 °C and yeast extract peptone dextrose (OXOID, UK) agar at 28 °C.

Cells (including murine macrophage RAW 264.7, human embryonic kidney 293 cell (HEK-293T), and hepatocellular carcinoma cell line HepG2) were obtained from the Stem Cell Bank at the Chinese Academy of Sciences (Shanghai, China).

LL-37 was synthesized from GL Biochem (Shanghai, China), and the antibiotic polymyxin B (PMB) were purchased from Beijing Solarbio Science & Technology Co., Ltd. (Beijing, China).

### 4.2. AMP Prediction and Truncated Peptide Synthesis

After obtaining the full-length cDNA sequences of Spampcin gene (details are presented in Supplementary Materials), the AMP possibilities of its deduced amino acid sequences were predicted by the antimicrobial peptide database CAMP_R3_, as well as the antimicrobial region within peptides. In CAMP_R3_, 4 different prediction methods are involved, including support vector machine (SVM), random forest (RF), artificial neural network (ANN), and discriminant analysis (DA), and the closer the value is to 1, the higher the probability of AMP. Interestingly, we found that both the Spampcin protein and its derived region from position 56th to 86th showed high AMP potential (Table 1), named Spampcin_56–86_ (Spa31 for short) (RRAAHGLLPRLRAPPPFHKRCVCLCRTAPPP). Afterwards, several truncated peptides derived from Spa31 were also selected for antimicrobial activity detection, including Spampcin_56–75_ (Spa20 for short) (RRAAHGLLPRLRAPPPFHKR), Spampcin_65–86_ (Spa22 for short) (RLRAPPPFHKRCVCLCRTAPPP), and Spampcin_65–78_ (Spa14 for short) (RLRAPPPFHKRCVC). Their AMP prediction results were summarized in Table 1. Four peptides were further chemically synthesized by GL Biochem (Shanghai, China) with a purity of >95%, and verified by high-performance liquid chromatography and mass spectrometry (Figures S3–S6). The total net charges and hydrophobic ratios of the four peptides were calculated by APD3. The 3D structures of four peptides were created using the Pep-Fold structure prediction tool for de novo peptides (https://mobyle.rpbs.univ-paris-diderot.fr/cgi-bin/portal.py#forms::PEP-FOLD3 (accessed on 26 September 2022)).

### 4.3. Antimicrobial Assay

The minimum inhibitory concentrations (MICs) and minimum bactericidal concentrations (MBCs) of four synthesized peptides were determined in triplicate on separate occasions following a previously described protocol [67]. Briefly, four peptides were serially diluted with Milli-Q water and placed on ice until use. All strains were harvested in logarithmic growth phase, washed with 10 mM Dulbecco’s phosphate buffered saline (DPBS, pH 7.4), and adjusted to a concentration of 1 × 10^6^ CFU mL^−1^ (bacteria) or 1 × 10^4^ cells mL^−1^ (mold spores) in Mueller-Hinton (MH) broth. After incubation with an equal volume of peptides for 24 h (bacteria) or 48 h (mold spores), MIC and MBC values were determined. The effect of Spa31 on the germination of mold spores was further observed by light microscope (Leica Dmi1, Wetzlar, Germany). Experiments were performed in triplicate and repeated at least three times independently.

### 4.4. Zone of Inhibition Assay

*S. aureus*, *P. aeruginosa*, and *E. coli* were selected to conduct the zone of inhibition assay as previously described [68] with some modifications. Briefly, exponentially growing bacteria were harvested and diluted to a concentration of 1 × 10^6^ CFU mL^−1^ in MH broth and 100 μL of bacterial solution were spread evenly on a sterile agar plate. Then 4 blank antimicrobial susceptibility discs (Bkmam, Changde, China) were pasted on the surface of the plate, and 20 μL of Spa31 or LL-37 at a concentration of 1 μg/μL were added to different discs. Meanwhile, 20 μL of Milli-Q water and 1 μg/μL of BSA at were used as controls. They were incubated overnight at 37 °C. The plate was observed the next day and the size of the inhibition zone was measured by a vernier caliper (Meinaite, Shanghai, China). Experiments were performed in triplicate and repeated at least twice independently.

### 4.5. Time-Killing Kinetics

*S. aureus* and *P. aeruginosa* were further selected to determine the bactericidal kinetics of Spa31 following a previous report [20]. Briefly, the final concentration of Spa31 was adjusted to 3 μM or 6 μM (1 × MIC or 1 × MBC for both bacteria). After incubating Spa31 with bacteria (prepared as described above for antimicrobial assay) for a period of time (within 30 min), bacterial suspensions were diluted and spread on nutrient broth plates at different time points and incubated at 37 °C for 24 h. Bacterial colony counts were recorded, and the bactericidal kinetic curves were drawn using GraphPad Prism 8.3.0 version (San Diego, CA, USA). Experiments were performed in triplicate and repeated at least three times independently.

### 4.6. Scanning Electron Microscope (SEM) Observation

The effects of Spa31 on *S. aureus* and *P. aeruginosa* were observed using SEM. The steps to prepare SEM samples refer to a previous study [17]. Briefly, 6 μM of Spa31 was incubated with the bacteria (1 × 10^7^ CFU mL^−1^) first. After incubation for a period of time (10 min or 30 min), they were fixed with pre-cooled 2.5% glutaraldehyde at 4 °C for 2 h, dehydrated in a series of concentrations of ethanol in a critical point dryer (EM CPD300, Leica, Wetzlar, Germany), coated with gold, and finally observed under a scanning electron microscope (Zeiss SUPRA 55, Oberkochen, Germany).

### 4.7. SYTO 9 and PI Staining Assay

The permeability of Spa31 on the bacterial inner membrane of *S. aureus* and *P. aeruginosa* was analyzed. The collection of exponential phase bacteria was adjusted to a final concentration of 1 × 10^7^ CFU mL^−1^. The SYTO 9/PI (propidium iodide) mixture was prepared following the instructions of the LIVE/DEAD^®^ *Bac*Light^TM^ Bacterial Viability Kit (Invitrogen, Waltham, MA, USA). Equal volumes of bacteria and Spa31 (diluted to a final concentration of 6 μM) were incubated at 37 °C for 30 min, followed by the addition of the dye mix and incubated at room temperature for 15 min in the dark. The fluorescence intensities of SYTO 9 and PI in bacteria were detected by flow cytometry (CytoFLEX, Beckman, Brea, CA, USA) and observed by confocal laser scanning microscope (CLSM, Zeiss Lsm 780 NLO, Oberkochen, Germany). FlowJo_v10.7.1 software (Brea, CA, USA) was used for data analysis. Experiments were performed in triplicate and repeated at least three times independently.

### 4.8. NPN, LPS Inhibition, ATP Release, ROS Production Assays

To further reveal the antibacterial mechanism of Spa31 against *P. aeruginosa*, various assays were designed, including NPN, LPS inhibition, extracellular ATP release, and ROS production assays. *P. aeruginosa* and Spa31 were prepared as described above. Experiments were performed in triplicate and repeated at least three times independently.

#### 4.8.1. NPN Staining Assay

The outer membrane permeability of Spa31 against *P. aeruginosa* was determined using the probe N-phenyl-1-naphthylamine (NPN) (Sigma-Aldrich, St. Louis, MO, USA) as previously described [43]. Briefly, the exponential growing *P. aeruginosa* was harvested, resuspended in HEPES buffer (5 mM HEPES, 5 mM glucose, pH 7.4), and diluted to 1 × 10^8^ CFU mL^−1^. NPN was then added to the diluted bacterial solution to a final concentration of 10 μM and placed in a 96-well black microplate at room temperature in the dark for 5 min. Afterwards, Spa31 was added to the mixture at final concentrations of 0, 3, 6, 12, and 24 μM. LL-37 (12 μM) and polymyxin B (PMB, 1 μg/mL) were used as controls. Fluorescence intensities (excitation 350 nm, emission 420 nm) were measured every 2 min (30 min total) in a microplate reader (Tecan, Männedorf, Switzerland).

#### 4.8.2. LPS Inhibition Assay

The exponentially growing *P. aeruginosa* was harvested and adjusted to 1 × 10^6^ CFU mL^−1^ in MH broth and added to a 96-well plate. Spa31 and *P. aeruginosa*-derived LPS (Sigma-Aldrich, Männedorf, Switzerland) were then added at final concentrations of 3 μM of Spa31 (1 × MIC) and 0, 3, 6, 12, 24 μg/mL of LPS. The plate was placed in a microplate reader and detected every 0.5 h for 24 h. The absorbance at 595 nm was recorded and the curve was plotted using GraphPad Prism 8.3.0 version (San Diego, CA, USA).

#### 4.8.3. ATP Release Assay

*P. aeruginosa* suspensions at 1 × 10^8^ CFU mL^−1^ in MH broth were incubated with a series of concentrations of Spa31 (0, 3, 6, 12, and 24 μM) at 37 °C for 10 min. Then, the mixture was centrifuged, and the supernatant was collected and subjected to analysis of the extracellular ATP using a GloMax^®^ 20/20 Luminometer E5311 (Promega, Madison, WI, USA) according to the instructions of the Enhanced ATP Assay Kit (Beyotime, Shanghai, China).

#### 4.8.4. ROS Production Assay

As described above, *P. aeruginosa* (1 × 10^8^ CFU mL^−1^) was prepared and incubated with different concentrations of Spa31 (0, 1.5, 3, 6, 12 μM) at 37 °C for 30 min. After incubation, the cells were collected by centrifugation and stained with 10 μM DCFH-DA probe (Nanjing Jiancheng Bioengineering Institute, Nanjing, China) at 37 °C for 45 min. The cells were then harvested, placed in a 96-well black microplate and subjected to a microplate reader for fluorescence intensities detection (excitation 488 nm, emission 525 nm). Meanwhile, the cells were analyzed by flow cytometry (CytoFLEX, Beckman, Brea, CA, USA) and FlowJo_v10.7.1 software (Brea, CA, USA) was used for data analysis.

### 4.9. Biofilm Inhibition Assays

The biofilm inhibition assays were carried out as previously described [69,70] with some modifications. Experiments were performed in triplicate and repeated at least three times independently.

#### 4.9.1. Biofilm Formation Inhibition Assay

Exponentially growing *P. aeruginosa* were harvested, adjusted to 1 × 10^8^ CFU mL^−1^ in MH broth and placed in a 96-well plate. A series of concentrations of Spa31 (0, 1.5, 3, 6, 12, 24, and 48 μM) were added and incubated at 37 °C for 24 h. After incubation, planktonic cells were removed, and the wells were gently washed 3 times. The plate was then placed in a 60 °C oven for 30 min to fix the biofilm and stained with crystal violet (0.1%, *w/v*). The absorbance at 595 nm were recorded using a microplate reader (Tecan, Männedorf, Switzerland).

#### 4.9.2. Mature Biofilm Inhibition Assay

Similarly, exponentially growing *P. aeruginosa* was harvested, adjusted to 1 × 10^6^ CFU mL^−1^ in MH broth, and placed in a 96-well plate for 24 h at 37 °C to allow biofilm maturation. Planktonic cells were removed, and the wells were gently washed 3 times. Then, Spa31 (at the final concentrations of 0, 1.5, 3, 6, 12, 24, 48 μM) and resazurin (at the final concentration of 0.1 mM) were added. The plate was incubated at 37 °C for 6 h and the absorbance at 560 nm and 620 nm were measured with a microplate reader (Tecan, Männedorf, Switzerland). The respiratory activity of cells in biofilms were evaluated by a modified resazurin assay as previously described [66].

### 4.10. Cytotoxicity and Hemolytic Activity Assay

#### 4.10.1. Cytotoxicity Assay

The cytotoxicity of Spa31 on cell lines (including RAW 264.7, HEK-293T, and HepG2 cells) was evaluated using the MTS method as previously described [15]. The cell lines used in the study were all mycoplasma-negative, which were purchased from the Stem Cell Bank at the Chinese Academy of Sciences and also confirmed by the mycoplasma test kit according to the manufacturer’s instructions (Solabio Life Science, Beijing, China) (Figure S7). Briefly, cells were seeded at 10^4^ cells well^−1^ in a 96-well plate overnight at 37 °C with 5% CO_2_. Then, the medium containing Spa31 was added at final concentrations of 0, 3, 6, 12, 24, and 48 μM and incubated for 24 h. Cell viability was determined following the instructions for the CellTiter 96^®^ AQueous Kit (Promega, Madison, WI, USA). Experiments were performed in triplicate.

#### 4.10.2. Hemolytic Activity Assay

The hemolytic activity of Spa31 on mouse erythrocytes was assessed as previously described [43]. Briefly, fresh mouse erythrocytes were collected, washed several times until the upper phase was clear, and adjusted to a concentration of 5 × 10^9^ cells mL^−1^. Then, Spa31 was added at the final concentration of 15.625, 31.25, 62.5, 125, 250 μg/mL (4.5, 9, 18, 36, 72 μM, respectively) and incubated at 37 °C for 1 h. PBS and 0.5% TritonX-100 treatment were used as negative and positive controls, respectively. Absorbance at 540 nm were recorded using a microplate reader (Tecan, Männedorf, Switzerland) and the hemolysis rates were calculated as previously described [43].

All animal procedures were carried out in strict accordance with the National Institute of Health Guidelines for the Care and Use of Laboratory Animals and were approved by the Animal Welfare and Ethics Committee of Xiamen University.

### 4.11. Evaluation of the In Vivo Infective Effect of Spa31 on Zebrafish D. rerio Infected with P. aeruginosa

To investigate the in vivo activity of Spa31, a zebrafish *D. rerio* bacterial infection model was used. After one week of temporary feeding in the laboratory, the fish were pre-injected intraperitoneally with *P. aeruginosa* at the final concentration of 3.7 × 10^6^ CFU fish^−1^. After 15 min of infection, Spa31 (5 μg fish^−1^ or 10 μg fish^−1^) was injected. And the control group was injected with the same volume of PBS. There were 24 zebrafish in each of the experimental groups and the control group. The survival rates of zebrafish were recorded at different time points and the survival curve was drawn with GraphPad Prism 8.3.0 version (San Diego, CA, USA). The independent assay was repeated at least twice.

All animal procedures were carried out in strict accordance with the National Institute of Health Guidelines for the Care and Use of Laboratory Animals and were approved by the Animal Welfare and Ethics Committee of Xiamen University.

### 4.12. Statistical Analysis

All data were presented as mean ± standard deviation (SD). All the statistical analyses were performed using GraphPad Prism 8.3.0 version (San Diego, CA, USA). For the extracellular ATP, ROS production, and biofilm inhibition assays, one-way ANOVA was employed to compare each treatment group with the control group. For the in vivo assay, survival comparisons were analyzed using the Log-rank (Mantel-Cox) test. Difference was considered as significant at *p* value < 0.05.

## 5. Conclusions

In summary, a new AMP, Spampcin_56–86_ (Spa31 for short), was identified from *Scylla paramamosain* and three truncated peptides derived from Spa31 were also found to exhibit potent antibacterial activity with different spectra. Among them, Spa31 showed the strongest antibacterial activity against a variety of clinical pathogens (such as *P. aeruginosa*, *E. coli*, *A. baumannii*, *S. aureus*, *L. monocytogenes*, and *E. faecalis*) with the MBC values ranging from 1.5 μM-6 μM and could kill *S. aureus* and *P. aeruginosa* within minutes. Spa31 interacted with LPS, and directly disrupted the integrity of the inner and outer cell membrane of *P. aeruginosa*, resulting in the release of ATP and the production of ROS, which might be the bactericidal mechanism of Spa31. In addition, Spa31 had a significant anti-biofilm effect against *P. aeruginosa*, not only on biofilm formation but also on mature biofilms. The antibacterial activity of Spa31 had good thermal stability, certain ion tolerance, and had no obvious cytotoxicity in vitro, and Spa31 exhibited an anti-infective effect in vivo which could significantly improve the survival rate of zebrafish *Danio rerio* infected with *P. aeruginosa*.

## Figures and Tables

**Table 1:** AMP probability of four synthetic peptides by CAMP_R3_ tools.

Name	Support Vector Machine	Random Forest	Artificial Neural Network	Discriminant Analysis
Spampcin	1.000	0.943	AMP	1.000
Spa31	0.978	0.873	AMP	0.930
Spa20	0.921	0.771	AMP	0.869
Spa22	0.912	0.794	AMP	0.974
Spa14	0.782	0.601	AMP	0.568

**Table 2:** Antimicrobial activity of four synthetic peptides.

Microorganism	CGMCC NO.	MIC ^a^ (μM)	MBC ^b^/MFC ^c^ (μM)			
		Spa31	Spa31	Spa20	Spa22	Spa14
**Gram-positive bacteria**						
*Staphylococcus aureus*	1.2465	1.5–3	3–6	12–24	6–12	>96
*Listeria monocytogenes*	1.10753	1.5–3	1.5–3	24–48	3–6	12–24
*Enterococcus faecalis*	1.2135	1.5–3	1.5–3	>96	3–6	>96
*Enterococcus faecium*	1.131	0–1.5	1.5–3	>96	3–6	>96
*Staphylococcus epidermidis*	1.4260	3–6	3–6	6–12	6–12	>96
**Gram-negative bacteria**						
*Pseudomonas aeruginosa*	1.2421	1.5–3	3–6	12–24	6–12	>96
*Escherichia coli*	1.2389	3–6	3–6	12–24	6–12	>96
*Acinetobacter baumannii*	1.6769	3–6	3–6	3–6	6–12	48–96
**Fungi**						
*Crytococcus neoformans*	2.1563	6–12	6–12	24–48	3–6	6–12
*Fusarium graminearum*	3.4521	1.5–3	1.5–3	12–24	12–24	12–24
*Fusarium solani*	3.5840	1.5–3	3–6	12–24	12–24	24–48
*Aspergillus niger*	3.316	12–24	12–24	12–24	12–24	24–48

^abc^ The values of MIC and MBC/MFC are expressed as the interval [a]–[b]. [a] is the highest concentration with visible microbial growth in the tested, and [b] is the lowest concentration with no visible microbial growth.

## Data Availability

The data presented in this study are available on request from the corresponding author.

## Supplementary Materials

The following supporting information can be downloaded at: https://www.mdpi.com/article/10.3390/ijms232113316/s1. References [11,71] are cited in the Supplementary Materials.

## Abbreviations

aa: amino acids; AMPs, antimicrobial peptides; ANN, artificial neural network; ATP, adenosine triphosphate; BSA, bovine albumin; cDNA, complementary DNA; CFU, colony-forming unit; CGMCC, China General Microbiological Culture Collection Center; DA, discriminant analysis; DCFH-DA, 2,7-Dichlorodi -hydrofluorescein diacetate; DNA, deoxyribonucleic acid; DPBS, Dulbecco’s phosphate buffered saline; hBD3, human β-defensin 3; LPS, Lipopolysaccharide; MBC, minimum bactericidal concentration; MFC, minimum fungicidal concentration; MH, Mueller-Hinton broth; MIC, minimum inhibitory concentration; NPN, N-phenyl-1-naphthylamine; ORF, open reading frame; PBS, phosphate buffered saline; PCR, polymerase chain reaction; PI, propidium iodide; PMB, polymyxin B; pI, isoelectric point; qPCR, real-time quantitative polymerase chain reaction; RACE, rapid-amplification of cDNA ends; RF, random forest; ROS, reactive oxygen species; SD, standard deviation; SEM, scanning electron microscope; SVM, support vector machine.
