# Supplementary material for: A Novel Antimicrobial Peptide Spampcin56–86 from Scylla paramamosain Exerting Rapid Bactericidal and Anti-Biofilm Activity In Vitro and Anti-Infection In Vivo
Source: Int J Mol Sci. 2022 Nov 1;23(21):13316. doi: 10.3390/ijms232113316 (PMC9653689; doi:10.3390/ijms232113316)
Supplement: Supplementary file 1 [file ijms-23-13316-s001.zip › ijms-1965401-supplementary.pdf]

Supplementary Materials

For

A Novel Antimicrobial Peptide Spampcin$_{56–86}$ from *Scylla paramamosain* Exerting Rapid Bactericidal and Anti-Biofilm Activity In Vitro and Anti-Infection In Vivo

Manyu Jiang [1,†], Roushi Chen [1,†], Jingrong Zhang [1], Fangyi Chen [1,2,3,*] and Ke-Jian Wang [1,2,3,*]

[1] State Key Laboratory of Marine Environmental Science, College of Ocean & Earth Sciences, Xiamen University, Xiamen 361102, China
[2] State-Province Joint Engineering Laboratory of Marine Bioproducts and Technology, College of Ocean & Earth Sciences, Xiamen University, Xiamen 361102, China
[3] Fujian Innovation Research Institute for Marine Biological Antimicrobial Peptide Industrial Technology, College of Ocean & Earth Sciences, Xiamen University, Xiamen 361102, China
* Correspondence: chenfangyi@xmu.edu.cn (F.C.); wkjian@xmu.edu.cn (K.-J.W.)
† These authors contributed equally to this work.

Animals, challenge and tissue collection

Mud crabs (*S. paramamosain*) were purchased from the Zhangpu Fish Farm (Fujian, China), and were allowed to acclimate for 3 days before experiments. Zebrafish was purchased from Xiamen Fish Farm (Fujian, China), and were allowed to acclimate for one week before experiments. Tissues were dissected using sterile tools, and flash frozen in liquid nitrogen and stored at -80 ℃ until use. To investigate the *in vivo* expression profiles of Spampcin, healthy male and female adult mud crabs (bodyweight 300 ± 30 g, n = 5) were dissected, and tissues including testis, anterior vas deferens, seminal vesicle, posterior vas deferens, ejaculatory duct, posterior ejaculatory duct, penis, ovaries, spermathecae, reproductive duct, muscle, thoracic ganglion, gills, brain, midgut, subcuticular epidermis, eye stalk, heart, hepatopancreas and stomach were collected. Hemocytes were isolated from the hemolymph as described previously [1]. For the *V. alginolyticus* challenge experiment, male crabs (body weight 300 ± 30 g, n = 5) were injected with *V. alginolyticus* (1×10^6 CFU crab^{-1}). For the *S. aureus* challenge experiment, male crabs (body weight 300 ± 30 g, n = 5) were injected with *S. aureus* (1×10^6 CFU crab^{-1}). Crabs injected with crab saline were set up as the control group. Tissue samples hepatopancreas were collected at 3, 6, 12, 24, 48 h and 72 h post-injection.

cDNA cloning

Following the manufacturer's instructions, total RNA of testis was extracted using Trizol™ reagent (Invitrogen, USA) and cDNA was generated using a PrimeScript™ RT reagent Kit with a gDNA Eraser Kit (Takara, China). The cDNA templates for 5′- and 3′- random amplification of cDNA ends (RACE) PCR were synthesized using a SMARTer® RACE 5′ /3′ Kit (Takara, China). Gene-specific primers were designed based on the partial sequences obtained from the transcriptome database established by our laboratory (Table S1). The amplified fragments were cloned into the pMD18-T Vector (Takara, China) and sequenced by Borui biotechnology Ltd. (Xiamen, China). The homology and similarity of the Spampcin cDNA sequence was analyzed using an online tool from the National Center for Biotechnology Information (NCBI, https://www. ncbi.nlm.nih.gov/). The theoretical isoelectric point and molecular weight were calculated by Compute pI/Mw Tool (https://web.expasy.org/compute_pi/).

Quantitative real-time PCR

Total RNA was extracted and cDNA was generated as described above. Quantitative real-time PCR (qPCR) was performed on a LightCycler480 (Roche Diagnostics) using FastStart DNA Master SYBR Green I (Roche Diagnostics). The tissue distribution of the Spampcin transcript was detected by absolute qPCR assay, and the responses of the Spampcin gene to different stimuli were measured by relative qPCR. For the relative qPCR assay, Sp-GAPDH (GenBank accession number: JX268543.1) was chosen as the reference gene and quantified to normalize the Spampcin expression. The primer sequences are listed in Table 1. Data were analyzed using the algorithm of the $2^{-\Delta\Delta Ct}$ method [2]. All data were presented as mean ± standard error of the mean (SEM). For absolute qPCR, one-way ANOVA was employed to compare differences of Spampcin gene expression in different tissues. For relative qPCR assay, a method of multiple t test (one per row) was used.

Thermal stability and ionic tolerance assay

The thermal stability and ionic tolerance of Spa31 against *S. aureus*, *P. aeruginosa* and *E. coli* were evaluated. For the thermal stability assay, the concentration of Spa31 was adjust to 3 μM (2 × MBC value for the three tested bacteria) and bacteria (1 × 10^6 CFU mL^{-1}) were prepared as described above. After treatment of Spa31 in a 100°C water bath for 10 min, 20 min, and 30 min, an equal volume of bacteria was added and placed in a 37 °C incubator. For the ionic tolerance assay, a series of concentrations of NaCl (10 mM, 20 mM, 40 mM, 80 mM, and 160 mM) were co-incubated with a mixture of Spa31 and bacteria. OD_{600} values were recorded at different time points (0 h, 12 h, 24 h, 36 h, 48 h) using a microplate reader (Tecan, Switzerland). All data were presented as mean ± SEM.

Mycoplasma testing

The cell lines (including RAW 264.7, HEK-293T and HepG2 cells) were purchased from the National Infrastructure of Cell line Resources of China and they were all confirmed to be mycoplasma-negative by the mycoplasma test kit according to the manufacturer's instructions (Solabio Life Science, China).

Table S1. Primers used in the study.

Primers	Sequence（5′-3′）
**cDNA cloning**	
Spampcin-F	ATGATAATCGTGGACATGG
Spampcin-R	CTATCTGGCCACCGCCAGC
Spampcin-3′-F1	CATGTCAGCGGTCACATGTTTCAC
Spampcin-3′-F2	ACATGTTTCACTTCCCTACGCT
Spampcin-5′-R1	GGACACTGGAGGGTGATGCT
Spampcin-5′-R2	CAGTTGGGACACGTCAGCAT
**qPCR**	
qPCR-Spampcin-F	TCGTGGACATGGTTGCAGGTG
qPCR-Spampcin-R	ACGGGACACTGGAGGGTGAT
GAPDH-F	ACCCATGTTTGTGTGTGGTG
GAPDH-R	ACAGTGGTCATGAGGCCCTG

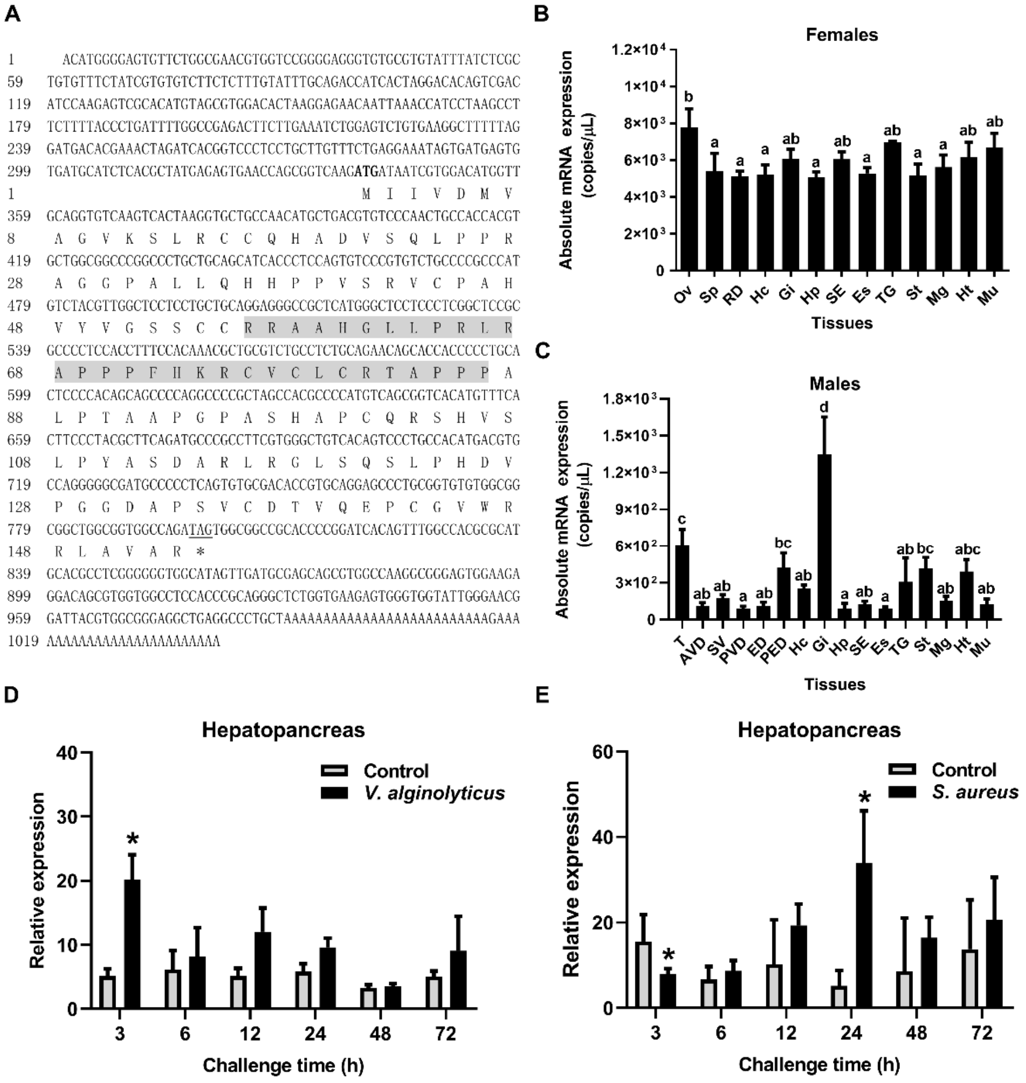

Figure S1. Sequences and expression profiles of Spampcin gene in *S. paramamosain*.
(**A**) cDNA and deduced amino acid sequences of Spampcin gene. The gray highlighted box represented the sequence of Spa31. Tissue distribution of Spampcin in females (**B**) and males (**C**) crabs (n = 5). The expression pattern of Spampcin in male hepatopancreas after *V. alginolyticus* (**D**) and *S. aureus* (**E**) challenge (n = 5). Differences in different tissues were indicated with the letter "a", "b" or "c". Significant difference between the control and bacterial challenge groups was indicated with asterisks, * $p < 0.05$. Abbreviations: Ov, ovaries; Sp: spermathecae; RD, reproductive duct; Hc, hemocytes; Gi, gills; Hp, hepatopancreas; SE, subcuticular epidermis; Es, eyestalk; TG, thoracic ganglion; St, stomach; Mg, midgut; Ht, heart; Mu, muscle; T, testis; AVD, anterior vas deferens; SV, seminal vesicle; PVD, posterior vas deferens; ED, ejaculatory duct; PED, posterior ejaculatory duct.

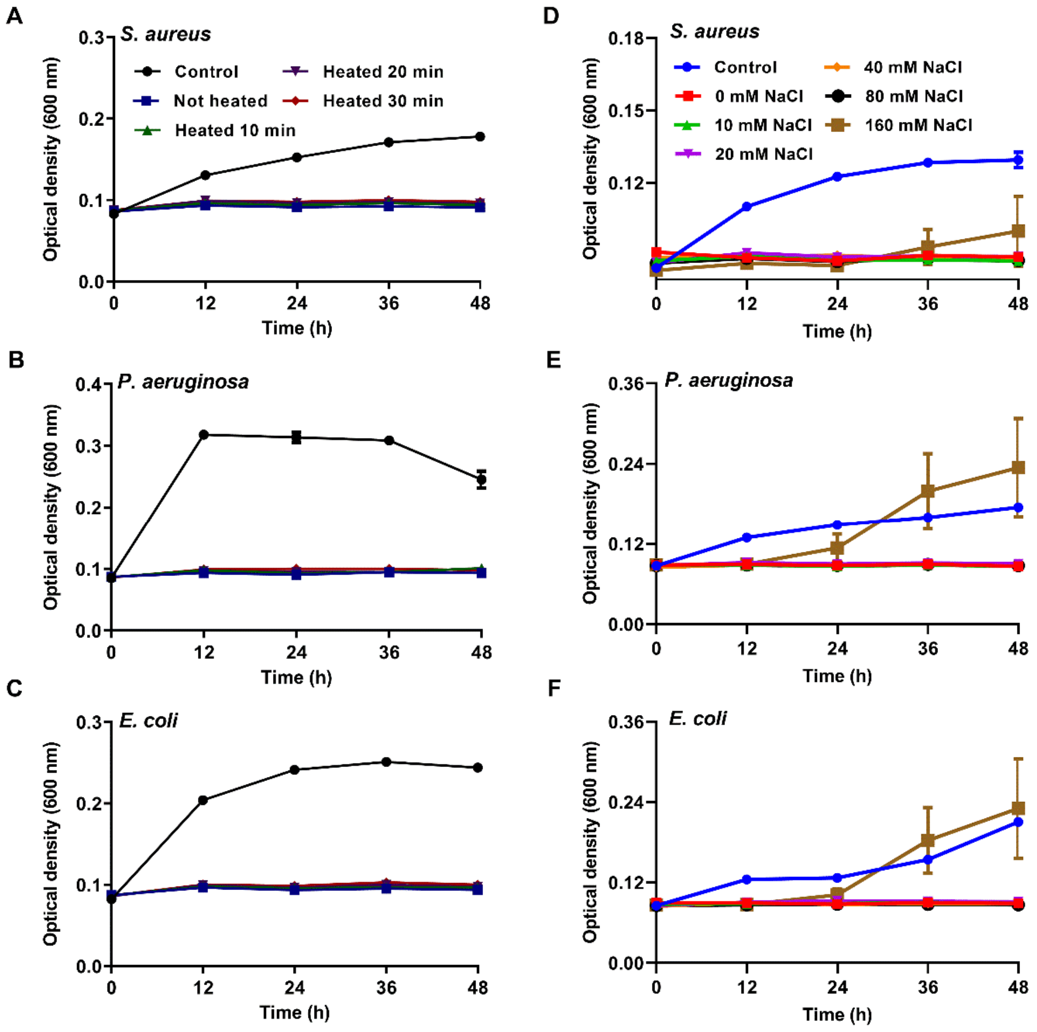

Figure S2. The thermal stability and ion tolerance of Spa31 against *S. aureus*, *P. aeruginosa* and *E. coli*.

The effect of 100°C water bath treatment on the antibacterial activity of Spa31 against *S. aureus* (**A**), *P. aeruginosa* (**B**) and *E. coli* (**C**). Effects of different concentrations of NaCl on the antibacterial activity of Spa31 against *S. aureus* (**D**), *P. aeruginosa* (**E**) and *E. coli* (**F**).

A

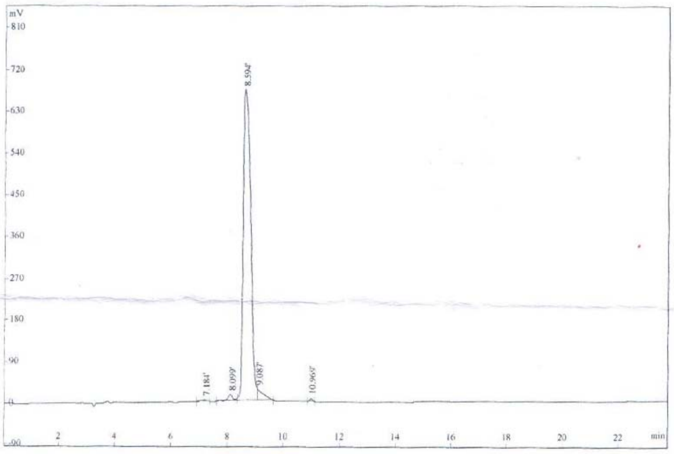

Rank	Time	Name	Conc.	Area	Height
1	7.184		0.3647	46400	3016
2	8.099		1.394	177355	13297
3	8.594		95.24	12116766	671807
4	9.087		2.656	337866	23034
5	10.969		0.3449	43881	6528

B

MASS SPECTROMETRY REPORT

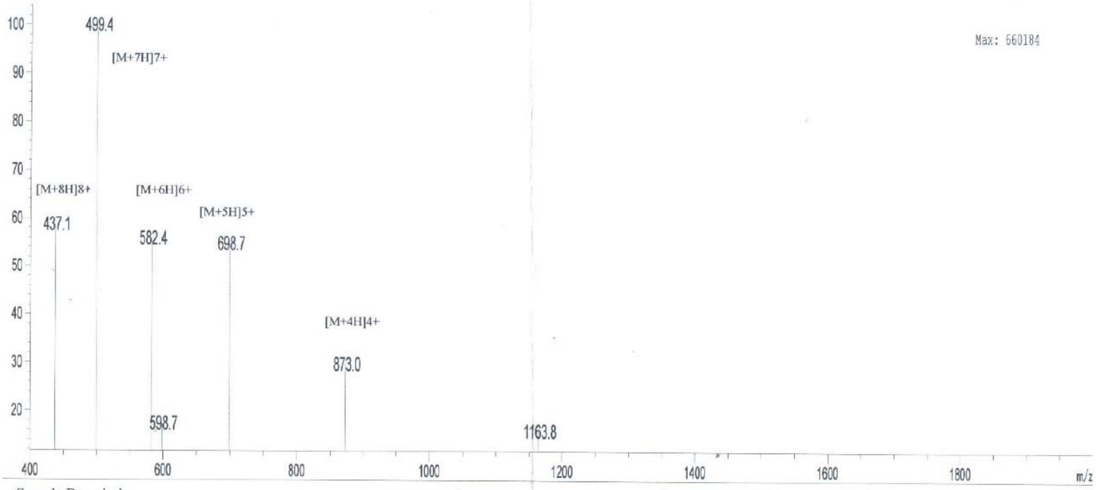

Sample Description		Instrument	Agilent-6125B		
Analyzed date:	2021-09-13	Probe:	ESI	Probe Bias:	+4.5kv
Analyst:	YU	Nebulizer Gas Flow:	1.5L/min	Detector:	1.5kv
Sample:	RP-31	CDL:	-20.0v	T. Flow:	0.2ml/min
M.W.:	3488.67	CDL Temp.:	250 °C	B. Conc.:	50%H2O/50%ACN
Lot. No.:	P210817-CL845495	Block Temp.:	200 °C		

Figure S3. The HPLC (A) and MS (B) data of Spa31.

A

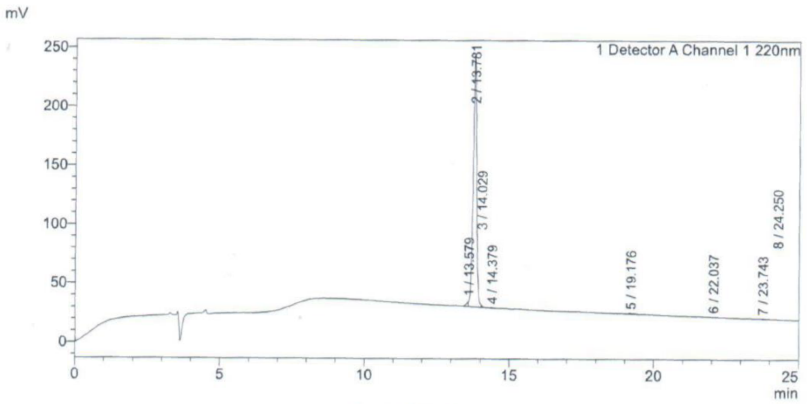

<Peak Table>

Detector A Channel 1 220nm

Peak#	Ret. Time	Area	Height	Area%
1	13.579	18391	3709	0.999
2	13.781	1808402	212456	98.206
3	14.029	6651	1919	0.361
4	14.379	1145	259	0.062
5	19.176	3029	492	0.164
6	22.037	1490	148	0.081
7	23.743	1197	202	0.065
8	24.250	1140	128	0.062
Total		1841446	219314	100.000

B

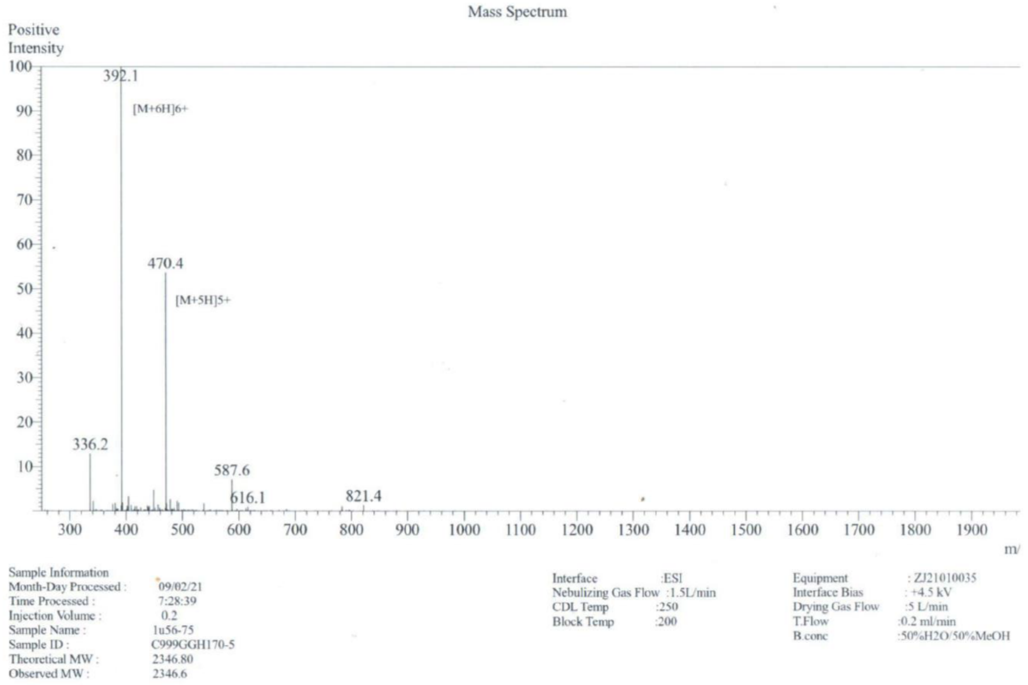

Figure S4. The HPLC (A) and MS (B) data of Spa20

A

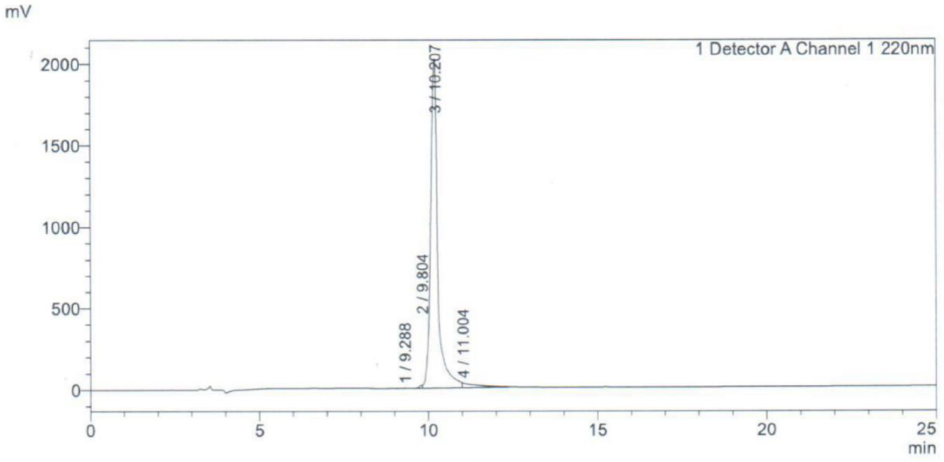

<Peak Table>

Detector A Channel 1 220nm

Peak#	Ret. Time	Area	Height	Area%
1	9.288	4471	1632	0.015
2	9.804	98259	17047	0.325
3	10.207	29249054	2021253	96.630
4	11.004	917441	29612	3.031
Total		30269225	2069544	100.000

B

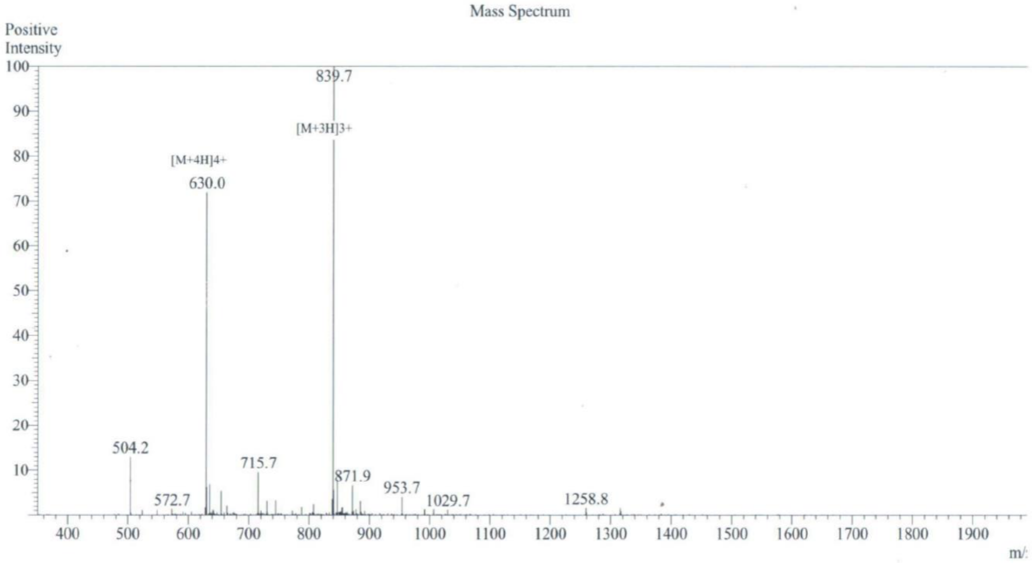

Sample Information		Interface	:ESI	Equipment	: ZJ21010035
Month-Day Processed :	08/25/21	Nebulizing Gas Flow	:1.5L/min	Interface Bias	: +4.5 kV
Time Processed :	21:55:16	CDL Temp	:250	Drying Gas Flow	:5 L/min
Injection Volume :	0.2	Block Temp	:200	T.Flow	:0.2 ml/min
Sample Name :	1u65-86			B.conc	:50%H2O/50%MeOH
Sample ID :	C999GGH170-3				
Theoretical MW :	2516.08				
Observed MW :	2516.1				

Figure S5. The HPLC (A) and MS (B) data of Spa22

A

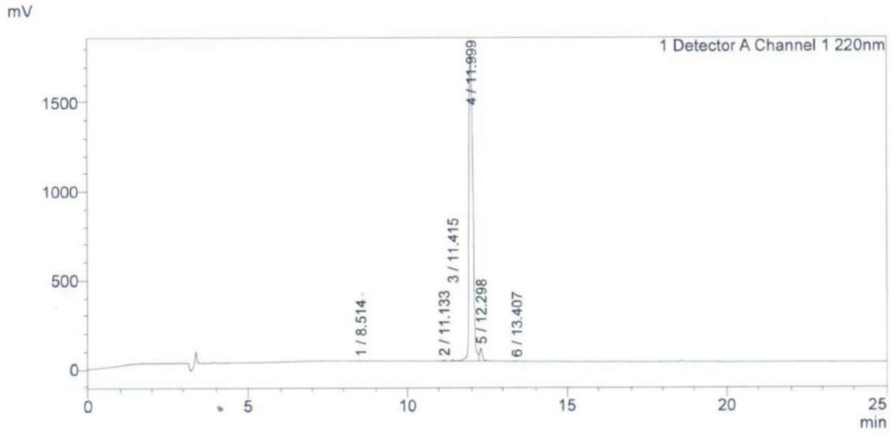

<Peak Table>

Detector A Channel 1 220nm

Peak#	Ret. Time	Area	Height	Area%
1	8.514	3606	391	0.025
2	11.133	34086	6351	0.236
3	11.415	53055	8104	0.367
4	11.999	13931316	1723354	96.339
5	12.298	431711	70755	2.985
6	13.407	6984	1122	0.048
Total		14460758	1810078	100.000

B

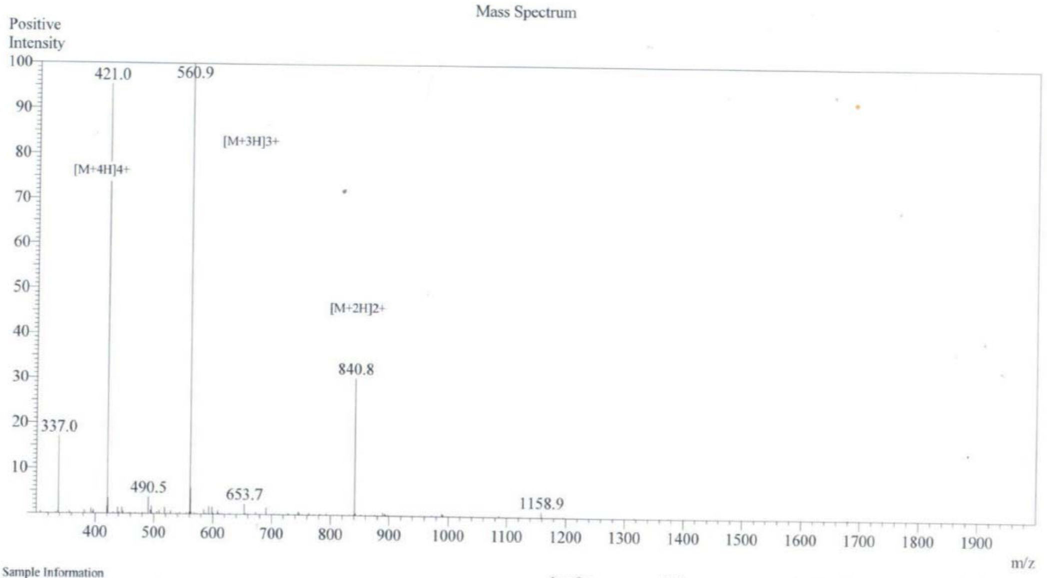

Sample Information
Acquired by : Gary
Month-Day Processed : 08/05/20
Time Processed : 01:16:42 PM
Injection Volume : 0.3
Sample Name : 1U（65-78）
Sample ID : C7038FG310-1
Theoretical MW : 1680.07
Observed MW : 1679.7

Interface :ESI
Nebulizing Gas Flow :1.5L/min
CDL Temp :250
Block Temp :200

Equipment : GK11010007
Interface Bias : +4.5 kV
Drying Gas Flow :5 L/min
T.Flow :0.2 ml/min
B.conc :50%H2O/50%MeOH

Figure S6. The HPLC (A) and MS (B) data of Spa14

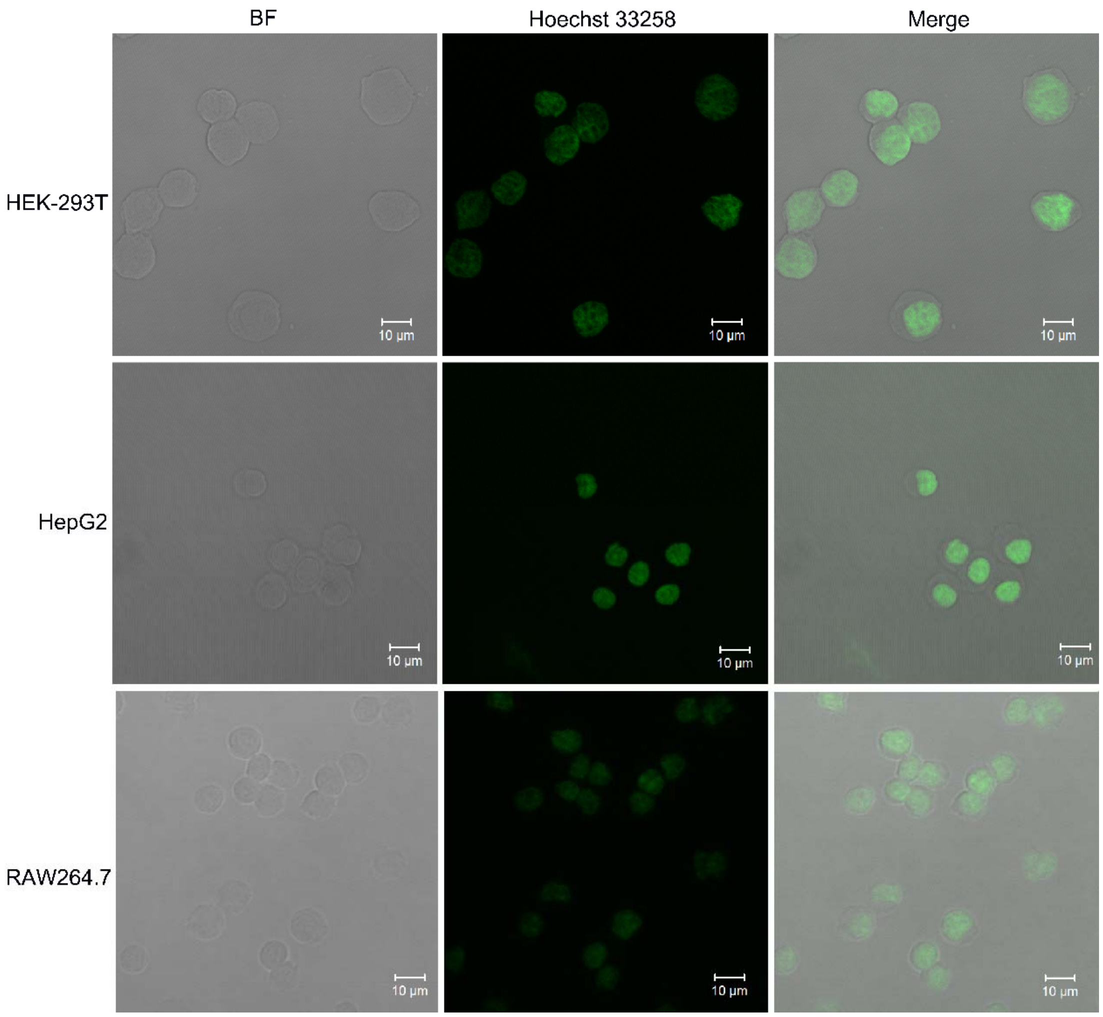

Figure S7. Mycoplasma testing of the three cell lines

References

1. Chen, F. Y.; Liu, H. P.; Bo, J.; Ren, H. L.; Wang, K. J., Identification of genes differentially expressed in hemocytes of *Scylla paramamosain* in response to lipopolysaccharide. *Fish Shellfish Immunol* **2010,** 28, (1), 167-77.
2. Livak, K. J.; Schmittgen, T. D., Analysis of relative gene expression data using real-time quantitative PCR and the $2^{-\Delta\Delta C_T}$ method. *Methods* **2001,** 25, (4), 402-408.
